# The feasibility and acceptability of an online mindfulness-based stress reduction program for chronic musculoskeletal pain: protocol for a pilot randomised controlled trial

**DOI:** 10.1186/s40814-025-01612-w

**Published:** 2025-03-15

**Authors:** Anita B. Amorim, Trudy Rebbeck, Nicholas T. Van Dam, Charlotte Johnstone, Claire Ashton-James, Nathalia Costa, Talia Barnet-Hepples, Matthew Jennings, Kathryn Refshauge, Evangelos Pappas

**Affiliations:** 1 Discipline of Physiotherapy, School of Health Sciences, Faculty of Medicine and Health, University of Sydney, Sydney, New South Wales Australia; 2 John Walsh Centre for Rehabilitation Research (Kolling Institute), Northern Sydney Local Health District, St Leonards, New South Wales Australia; 3 Contemplative Studies Centre, Melbourne School of Psychological Sciences, Faculty of Medicine, Dentistry and Health Sciences, University of Melbourne, Melbourne, Victoria Australia; 4 Department of Anaesthesia, Royal Prince Alfred Hospital, Sydney Local Health District, Sydney, New South Wales Australia; 5 Pain Management Research Institute, Faculty of Medicine and Health, Kolling Institute, University of Sydney, Sydney, New South Wales Australia; 6 South Western Sydney Local Health District, Sydney, New South Wales Australia; 7 School of Health and Biomedical Sciences, Royal Melbourne Institute of Technology, Melbourne, Victoria Australia

**Keywords:** Chronic musculoskeletal pain, Mindfulness-based stress reduction, Clinical trial, Pilot study, Feasibility

## Abstract

**Background:**

Chronic musculoskeletal pain conditions affect millions of people worldwide and place a significant burden on individuals and the healthcare systems. Managing chronic musculoskeletal pain requires a multidisciplinary approach that considers biological, psychological, and social factors. However, access to multidisciplinary pain care is challenging, and long wait times can lead to increased stress and health deterioration. Mindfulness-based stress reduction (MBSR) is a mind-body approach developed specifically to reduce the distress of living with chronic conditions, such as chronic musculoskeletal pain. This study proposed a novel approach by offering an online MBSR program to patients on waitlists to attend a multidisciplinary pain clinic in Australia’s public healthcare system that could improve health outcomes. The study aims to assess the feasibility, acceptability, and potential efficacy of this approach using a pilot study design with a mixed-methods approach.

**Methods:**

This is a mixed-methods pilot randomised controlled trial with an embedded qualitative study. Participants will be recruited from waitlists at two multidisciplinary pain management centres within the Sydney Local Health District in New South Wales, Australia. This pilot trial will randomly assign 32 individuals with chronic musculoskeletal pain to either an online MBSR group or a usual care control group. Feasibility outcomes, patient-reported outcomes, adherence to mindfulness practice, and adverse events will be assessed using validated questionnaires. Semi-structured interviews will be conducted with participants in the MBSR group to explore their experiences and evaluate acceptability, and barriers and facilitators of engagement with the intervention.

**Discussion:**

This pilot study will evaluate a novel approach to integrating MBSR into the Australian public healthcare system as a mechanism for providing support to individuals with chronic musculoskeletal pain who are waitlisted for a multidisciplinary pain management program. Findings from this study will indicate the feasibility, acceptability, safety, and preliminary efficacy of this approach in terms of patient-reported outcomes to guide the design of future large-scale clinical trials.

**Trial registration:**

This trial was prospectively registered in the Australian New Zealand Clinical Trials Registry (ACTRN12622000822785).

**Supplementary Information:**

The online version contains supplementary material available at 10.1186/s40814-025-01612-w.

## Background

Chronic musculoskeletal pain is a significant public health problem affecting millions worldwide [1], causing widespread suffering for individuals and substantial costs for society [2]. The 2019 Global Burden of Disease (GBD) report illustrates the staggering scale of this problem, reporting that over 1.7 billion individuals worldwide are living with musculoskeletal pain, including neck and lower back pain [3]. Musculoskeletal pain conditions collectively contribute to 149 million years lived with disability (YLD), constituting nearly 17% of the global disease burden. This ranks musculoskeletal pain as the predominant YLD contributor on a global scale [3]. In Australia alone, the financial cost of chronic musculoskeletal pain is estimated at $55 billion annually, incorporating healthcare utilisation, lost productivity, carer burden, and reduced quality of life [4, 5]. Despite treatment advances [6, 7], the prevalence and burden of chronic musculoskeletal pain have increased over time [8], and are expected to continue to increase over the coming decades [9, 10].

Chronic musculoskeletal pain is well-recognised as a multifaceted condition, characterised by a complex interplay of biological, psychological, and social factors, making it challenging to manage [11]. Individuals living with chronic musculoskeletal pain often report high levels of pain-related emotional distress [12–14]. Best practice guidelines recommend integrating physical and psychological interventions for managing chronic pain, often delivered by a multidisciplinary team [15]. This model is an established standard of care provided in public tertiary pain management centres in Australia and is based on specialised assessment and treatment processes, including a multidisciplinary team approach and group pain management programs [16]. However, wait times to access such services often far exceed the recommended 6-month maximum wait, with the most disadvantaged individuals experiencing up to 3 years in the waitlist [17]. Long delays in access to treatment can exacerbate distress and, consequently, physical and psychological deterioration [18]. While system-level strategies are required to reduce waitlist times, we can develop strategies to mitigate the distress and potentially improve the outcomes of patients who are on waitlists.

Mindfulness-based stress reduction (MBSR) is a mind-body training program developed specifically to address the distress associated with long-term conditions, such as chronic pain [19]. The MBSR program combines mindfulness meditation, body awareness, yoga, and exploration of patterns of behaviour, thinking, feeling and action [20]. By engaging participants holistically on multiple levels (i.e. physiological and emotional regulation, cognitive reappraisal, behavioural change, engagement with values, and social connection), MBSR has been shown to significantly reduce pain [21, 22], improve symptoms of anxiety, and depression and improve quality of life, in people with various chronic conditions [23], including chronic musculoskeletal pain [22–24]. MBSR is typically delivered as a face-to-face, 8-week program, with weekly 2.5-h group sessions, and an expectation of daily practice by participants [20]. However, recent evidence has shown that online delivery of MBSR is comparably effective to in-person delivery [25]. An online delivery mode allows participants to complete the program without the need to travel to a single location, which can pose logistical and financial challenges [26].

Despite evidence supporting the efficacy of MBSR in improving patient-reported outcomes for chronic pain [21], availability to patients in Australia remains limited. MBSR programs are primarily offered as privately funded courses, which are costly thereby excluding a large proportion of patients with chronic musculoskeletal who cannot afford it [27]. Addressing this barrier to access calls for the incorporation of MBSR programs into the public health system [28]. This shift could improve access to a mind-body approach that can improve the well-being of patients with chronic musculoskeletal pain, thereby mitigating health inequities. However, implementing a novel intervention in health services requires significant and planned changes, and the first step is to test its practicality [29]. In this context, pilot studies play a crucial role in testing and refining the research methods and procedures prior to a fully powered clinical trial. Pilot trials further assist in identifying potential barriers and facilitators related to the intervention, enable assessment of the feasibility of data collection and analysis, and by uncovering any issues early on, guide necessary adjustments that improve the quality and efficiency of fully powered clinical trials [30].

Our study, therefore, aims to (1) evaluate the feasibility of an online MBSR program for managing chronic musculoskeletal pain within the public health care system in Australia; (2) identify barriers and facilitators related to the online MBSR program for people with chronic musculoskeletal pain; and (3) determine the preliminary efficacy and safety of the online MBSR program compared to usual care on patient-reported outcomes.

## Methods

### Study design

This is a pilot randomised controlled trial (RCT) with an embedded qualitative study. We will follow the first two steps in the Sax Institute’s Translational Research Framework [31] to investigate the feasibility and preliminary efficacy of an online MBSR program for chronic musculoskeletal pain. This Translational Research Framework serves to bridge the gap between research and real-world implementation by systematically addressing barriers at different stages of translation. This study specifically focuses on two key translational steps: (1) T1 (feasibility testing): assessing the feasibility of the intervention in a controlled setting, including recruitment, adherence, and preliminary engagement outcomes; and (2) T2 (clinical research): assessing the preliminary efficacy of the intervention to determine its potential benefits. These steps provide a structured approach to evaluating the feasibility and preliminary efficacy of the online MBSR program for individuals with chronic musculoskeletal pain [32].

This study employs an exploratory sequential mixed-methods design, in which quantitative data will be collected first, followed by qualitative interviews to provide further insights into participants’ experiences. This approach enables the identification of key trends in the quantitative data, which can then be explored in greater depth through qualitative methods. The quantitative component will assess recruitment rates, intervention adherence, patient-reported outcomes, and adverse events, providing objective feasibility data. The qualitative component, gathered through semi-structured interviews, will explore participants’ experiences with the program, including barriers and facilitators to engagement, intervention acceptability (e.g. content, complexity, delivery, and areas for improvement), and key factors influencing future implementation and scalability. This integrated approach ensures a comprehensive evaluation of both feasibility and user experience, informing the design of a larger-scale trial. To ensure clarity and transparency in reporting, study findings will adhere to the CONSORT 2010 statement: extension to randomised pilot and feasibility trials [33]. The qualitative component will be reported following the Consolidated Criteria for Reporting Qualitative Research (COREQ): a 32-item checklist for interviews and focus groups [34].

### Participants’ inclusion and exclusion criteria

#### Pilot RCT

A total of 32 individuals with chronic musculoskeletal pain will be randomised to either an online MBSR group (*n* = 16) or a usual care control group (*n*= 16). This sample size has been determined based on feasibility considerations and recommendations for pilot studies. A detailed justification is provided in the Sample Size section (page 16). We will include people that (i) are 18 years or older; (ii) have chronic primary or secondary musculoskeletal pain as described by the IASP (International Association for the Study of Pain) classification of chronic pain for ICD-11 (i.e. “pain located in the muscles, bones, joints, or tendons”) [35]; (iii) are proficient in English (i.e. able to read and understand the consent form, participant information sheet, and study materials without the need for assistance from a translator); (iv) are able to access the internet via a computer (desktop or laptop) or a smartphone. We will exclude people who (i) have pain from serious pathologies such as fractures or cancer; (ii) have self-reported active or uncontrolled mental illness (i.e. severe depression, active suicidality, bipolar disorder, and schizophrenia that is not managed by a health care professional—psychologist, psychotherapist, psychiatrist); (iii) have a history of an unexplored, untreated traumatic experience or adverse childhood events assessed by an experienced clinician during the telephone screening process; or (iv) are judged at the investigator’s discretion as being unsuitable to participate (e.g. inability or unwillingness to attend the weekly group sessions).

#### Qualitative study

Participants from the intervention group (MBSR group) participating in the pilot RCT will also be invited to participate in the embedded qualitative study, which will use in-depth semi-structured interviews to explore the participant’s experience in the MBSR program. We will strive to recruit a diverse sample of participants covering a broad population, including people from varied occupation status and cultural backgrounds, age groups, and genders.

### Recruitment

#### Pilot RCT

##### Pre-screening

We will recruit individuals with chronic musculoskeletal pain who are on the waitlist to receive care at the pain management centres at Royal Prince Alfred Hospital (RPAH) and the Concord Repatriation General Hospital (CRGH) in the Sydney Local Health District (SLHD) in Sydney, New South Wales (NSW), Australia. The average time spent on the waiting list to access the Pain Management Centre at RPAH and CRGH varies from 6 to 12 months.

The Clinical Nurse Consultants at the RPAH and CRGH pain management centres will call individuals on the waitlists and inform them about the study. They have received training in describing the study and answering questions related to the study protocol from the Coordinating Principal Investigator. The Clinical Nurse Consultants have also been provided with a script to use during the telephone calls. If individuals are interested in learning more about the study, the Clinical Nurse Consultants will ask permission to (a) share their name and contact information with the research team; and (b) send them an email containing a study advertisement and a Participant Information Sheet that describes the study’s eligibility pre-screening process. The recruitment email also contains a link that will take them to a pre-screening Consent Form and a short pre-screener survey created with the Research Electronic Data Capture (REDCap), which will contain some questions regarding their eligibility for joining the study. Where permission is given, after each phone call, the Clinical Nurse Consultants will share the name, phone number and email address of the interested potential participants with the research team, via a form on REDCap.

If a potential participant does not answer the Clinical Nurse Consultant’s phone call, they will be sent an SMS that will inform them about the study. The SMS will contain a URL link that will take them to an online copy of the Participant Information Sheet, the pre-screening Consent Form, and the short pre-screener survey. The SMS will be sent using a programmable messaging API (Application Programming Interface) from Twilio that is integrated with REDCap. Potential participants can reply ‘STOP’ to the SMS to opt out of any future communications about the study.

Potential participants who expressed interest in the study over the phone with one of the Clinical Nurse Consultants who did not complete the pre-screener survey will be sent a reminder email 3 to 5 days after the initial email. The reminder email will also mention that a research investigator may contact potential participants on their provided phone number to see if they have received this email and if they are still interested in learning more about the study.

If potential participants do not complete the pre-screener survey after the reminder email, a research investigator will call them 1 week after the initial contact from the Clinical Nurse Consultants to ask if they have received the email and if they are still interested in learning more about the study. This will be the last attempt to contact the participants. No further contact will be made if they are not interested. During the call, if they remain interested in the study, the research investigator will direct the potential participants to the email or re-send the email with the Participant Information Sheet, Consent Form and the pre-screener survey. Potential participants will also have the opportunity to ask questions about the study during the phone call.

If a potential participant who received a recruitment SMS and did not opt out of future communications about the study does not complete the pre-screener survey, they will be sent a follow-up SMS 5 days after the initial SMS. This will be the last attempt to contact the participants. No further contact will be made if they are not interested.

If eligible after completing the pre-screener survey, potential participants will receive a phone call from an experienced MBSR teacher and psychotherapist to screen for mental health concerns. During the phone call, the MBSR teacher will ask eleven questions to screen for any severe and uncontrolled mental health condition or a history of an unexplored, untreated traumatic experience. If eligible after completing the mental health screen, potential participants will be reminded that if they agree to participate in the study, they have a 50% chance of being in either the MBSR group or the usual care control group.

Participants in the usual care group will continue to receive the care that patients usually receive while they are on the waitlist to attend the pain management centres at the RPAH and CRGH. Potential participants will also be asked their preferred day and time to attend the weekly 2.5-h MBSR group sessions, in case they agree to enrol in the study and are allocated to the MBSR group. If ineligible, individuals will be provided with the reason why they are not eligible for the study. All ineligible individuals will be given the contact details of the Coordinating Principal Investigator, who can clarify any questions they may have. They will be informed they will remain on the waitlist to receive care at the RPAH or the CRGH pain management centres. If individuals are distressed because they are ineligible for the study, they will be referred to the Pain Specialist and Site Principal Investigator from the relevant Pain Management Centre, or if unavailable, the relevant Clinical Nurse Consultant, for support. If necessary, the Clinical Nurse Consultant will refer the participant to the Clinical Psychologist available on the day.

### Enrolment

**Fig. 1:**
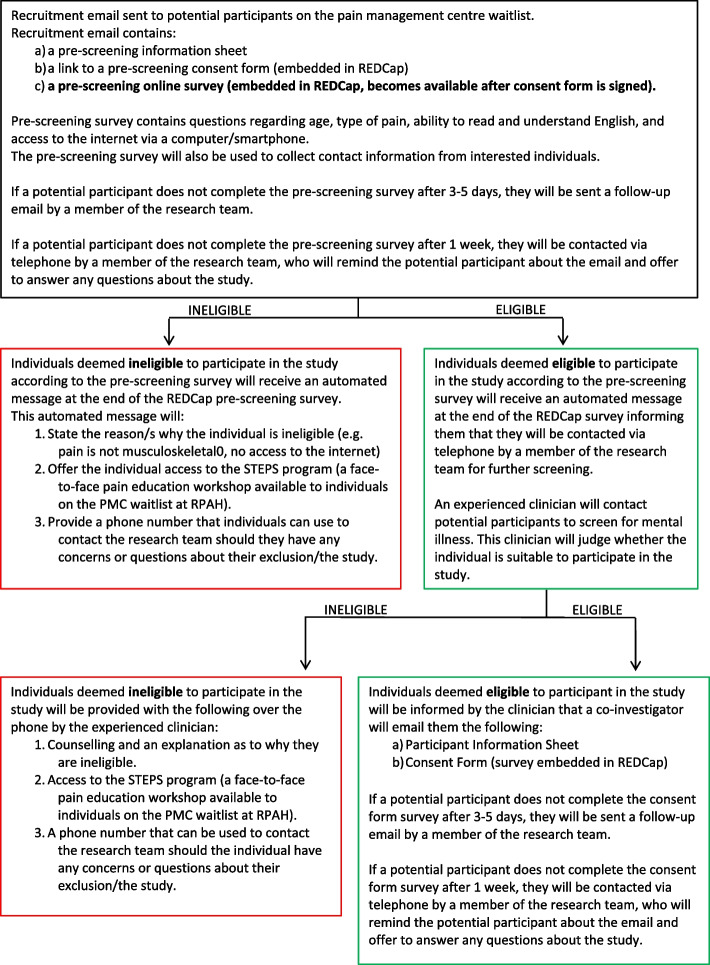
Screening flowchart

Potential participants who are deemed eligible to join the pilot RCT after the pre-screening process, and who indicate an interest in participating in the pilot RCT, will be sent the pilot RCT Participant Information Sheet via email to keep. Potential participants can take their time to think and decide. If they agree to join the RCT, they will be asked to sign a consent form embedded into REDCap. A link to the consent form will be sent via email. Potential participants who do not complete the electronic consent form will be sent a follow-up email 3 to 5 days after the initial email. The reminder email will also mention that a research investigator may contact potential participants on their provided phone number to check receipt of this email and confirm that they are still interested in participating in the study. If a potential participant does not complete the electronic consent form after the follow-up email, a research investigator will call them 1 week after the initial email to determine whether they have received the email and whether they are still interested in participating in the study. This will be the last attempt to contact the participants. The screening process is illustrated in the screening flowchart (Fig. 1). Should participants volunteer, the research investigator will emphasise that they are free to withdraw from the study at any time without penalty.

Group allocation

After eligibility is confirmed, participants will be randomly assigned to either the online MBSR intervention or the control group using a computer-generated randomisation sequence with a 1:1 allocation ratio. An independent researcher not involved in recruitment will generate the randomisation sequence to minimise bias. Randomisation will occur after baseline data collection to prevent allocation bias.

#### Qualitative study

All participants completing the MBSR program as part of the pilot RCT will be purposively invited for in-depth interviews after completing the program. Research investigators will contact MBSR program participants to invite them to take part in the interviews after the program is finished. Potential participants interested in participating in the interviews will receive the Participant Information Sheet and Consent Form via email.

### Intervention

1. Participants will be randomised into one of two parallel groups: (1) an online live facilitated MBSR group or (2) a usual care control group.Participants in the online live facilitated MBSR group will participate in an 8-week intervention involving weekly 2.5-h online group sessions and one online half-day workshop. This 6-h workshop, called “The Day of Mindfulness”, will be scheduled between the sixth and seventh week, and is held in silence with only the instructor speaking. This “retreat” will provide participants with an opportunity to deepen what they learn during the weekly online sessions. Participants will be encouraged to practice mindfulness daily throughout the 8-week period. In addition, participants will receive access to a 100-page coursebook, and a mobile application called *Openground Mindfulness Training* which has pre-recorded guided meditation practices that participants can use throughout the program. Patient data and app usage data will not be sent to the mobile application developer. Participants will also receive ongoing individual support from the MBSR teacher, as necessary.

Mindfulness is defined as non-reactive awareness of the present experience, including body sensations, internal mental states, thoughts, emotions, impulses and memories [20]. Mindfulness meditation is a mind training approach that cultivates cognitive control, emotion regulation and acceptance (i.e. nonreactivity), which leads to reduced stress and increased well-being [20]. During the online MBSR program, participants will meet once a week for 2.5 h via an online platform to practice mindfulness meditation and body awareness. During this time, the participants will also interact with each other through discussions facilitated by a skilled, certified MBSR teacher. Our proposed mindfulness intervention is based on MBSR, but it has been adapted for individuals with chronic pain. This program integrates psychoeducation on the latest pain neuroscience based on the evidence-based Explain Pain program [36, 37]. Each week, the teacher presents the theoretical underpinnings of the mindfulness training and the application of self-regulatory skills and encourages dialogue and reflection on distinct topics as described in Table 1 below.

(2) Participants in the usual care control group will receive the type of care that is usually provided to individuals who are on the waitlist to attend the pain management centres at the RPAH and CRGH. This care often includes accessing brief pain education. This may be accessed through the STEPS program at the RPAH, the DIB program at the CRGH, or through My Pain Hub, a passive online evidence-based resource for musculoskeletal pain. Participants in this group will also be offered access to a passive online evidence-based educational resource for musculoskeletal pain developed by our team *My Pain Hub* (https://www.mypainhub.com). *MyPainHub* has been co-designed and developed by clinicians managing and people with musculoskeletal pain and tested in a recent RCT [38]. The patient pages contain accurate advice, information, and self-directed exercises for common musculoskeletal conditions.

**Table 1:** Adapted from the curriculum guide for MBSR

Week	Topic	Description
1	Recognising the present moment	Safety and guidelines are established. Participants get an overview of the pain science and an introduction to mindfulness theory and practice.
2	Perception and making sense of our world	Participants begin to explore how their physical and emotional experiences can be impacted by how they perceive internal and external factors. They begin to explore how the brain creates pain as an output from danger signals.
3	Learning about pain and reactivity	Participants deepen practice and learn to approach bodily sensations with a simple appraisal of how pleasant, unpleasant or neutral they are. They learn about how pain and tissue damage are poorly related.
4	Investigating stressful experiences	Participants learn to approach difficult experiences and build emotion regulation skills through education and practice. They learn how their own meanings of safety and danger can impact their pain experience.
5	Thoughts, emotions and language	This session emphasises the capacity of participants to adapt more rapidly and effectively to everyday challenges and stressors. Participants learn how their thoughts and language can impact their pain experience. They develop more safety and confidence in relation to their inner experience.
6	Relationships and kindness	Participants actively cultivate kindness for themselves and others, enhancing resilience and connection. They learn about the complexity of the pain experience and how they can enhance their responsiveness to their own distress by allowing and letting go.
7	Values and action	Participants are asked to purposefully reflect on lifestyle choices that are adaptive and self-nourishing and those that are maladaptive and self-limiting to integrate mindfulness practice more fully and personally into their daily lives.
8	Making mindfulness a part of your life	Participants reflect on the process, celebrate their successes and grieve their disappointments. They reflect on strategies and intentions for continuing to integrate what they have learned into their lives.

### Outcome measures

#### Pilot RCT

##### Feasibility outcomes

Feasibility outcomes will include recruitment rate, retention rate, suitability of data collection methods, and identification of successful recruitment methods. Records will be kept of the number of individuals screened for eligibility, the number eligible and invited to participate, and the number that consent to participate. We will also record reasons for not entering the study and reasons for dropping out. Our pilot trial will be deemed feasible if at least 80% of the participants recruited for our pilot trial remain in the study until its completion. Our progression criteria for the trial will follow the traffic light system proposed by Avery and colleagues [39], where achieving green (go)—80% retention rate—indicates that the criteria have been met, and the trial should proceed, achieving amber (amend)—60% retention rate—indicates that some changes should be made to the larger trial and achieving red (stop)—40% retention rate—indicates that the investigators should not move forward with the larger trial. To keep a record of the issues faced during the pilot trial, we will maintain a document called ‘lessons learned’ that details the problems encountered, the attempts made to resolve them, and whether they worked or not [40].

##### Patient-reported outcome measures (PROMs)

**Fig. 2:**
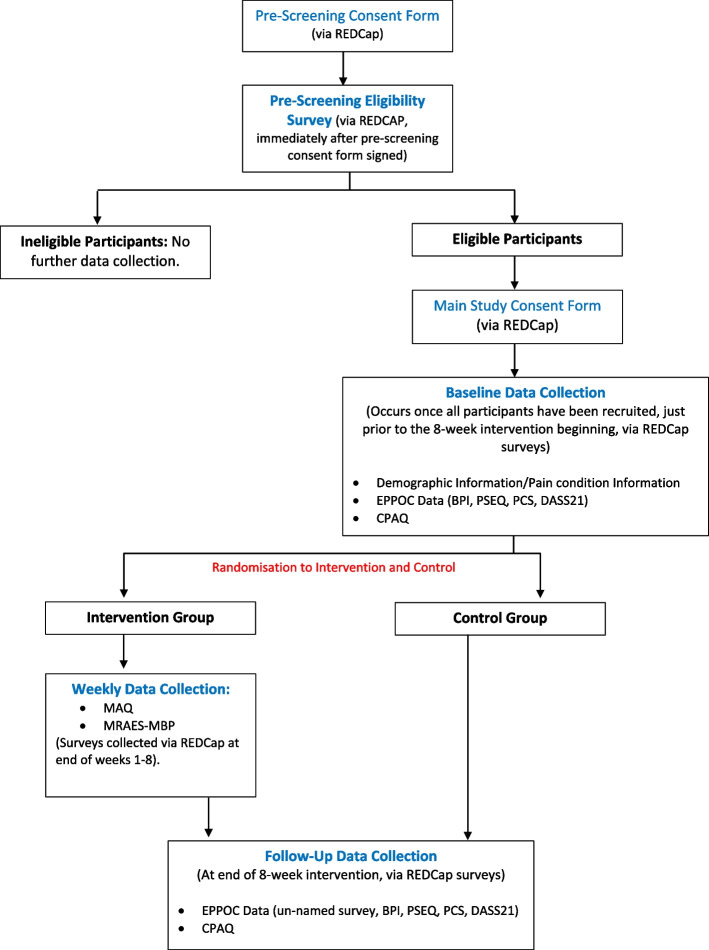
Data collection flowchart

Upon entry to the study, prior to randomisation, we will collect demographic information, including age, gender, race, ethnicity, education level, and postcode (to be used as a proxy for socioeconomic status). We will also collect information about the participant’s general health status and pain condition, including the number of years they have lived with chronic pain and their primary sources or locations of pain. Patient-reported outcome measures will be collected upon entry to the study prior to randomisation, and at the conclusion of the online MBSR program, to assess preliminary pre- to post-intervention changes. We will use the validated and widely used patient-reported outcome measures that make up the electronic Persistent Pain Outcomes Collaboration (ePPOC). The ePPOC is a resource for pain researchers and clinicians, which was established in NSW in 2013, to evaluate and assist in improving outcomes and services for people experiencing chronic pain [41]. The ePPOC measures pain and disability using the Brief Pain Inventory (BPI) [42], cognition using the Pain Self-Efficacy Questionnaire (PSEQ) [43], pain catastrophising using the Pain Catastrophising Scale (PCS) [44], mood using the Depression, Anxiety and Stress Scale (DASS-21) [45], health care utilisation and medication. These measures are recommended and used in pain management centres within Australia and New Zealand. We will also measure chronic pain acceptance using the Chronic Pain Acceptance Questionnaire (CPAQ) [46]. The CPAQ measures two aspects of chronic pain acceptance - activity engagement and pain willingness. Participants will be emailed a link to these questionnaires, which will be embedded into a web-based application (REDCap). The data collection process is illustrated in Fig. 2.

##### Adherence to the mindfulness practice

Adherence to the suggested daily mindfulness practices throughout the 8-week intervention will be measured weekly via REDCap, using an online version of the Mindfulness Adherence Questionnaire (MAQ) [47]. The MAQ is a 12-item self-reported questionnaire that captures adherence to the mindfulness practice within the past week. It was designed to assess regular and sustained practice in attentional focus and non-judgmental acceptance (i.e. quantity, quality, subtype of practice). The first two items measure the frequency and average duration (in minutes) of formal practices. The remaining 10 items measure the quality of formal practice and informal practice. Items are scored on a 7-point Likert scale ranging from 0 (never) to 6 (always), with higher total subscale scores reflecting higher practice quality.

##### Adverse events

The Meditation-Related Adverse Effects Scale - Mindfulness-Based Program version (MRAES-MBP) [48], will be used to collect data regarding adverse events. An email with a link to the MRAES-MBP embedded in REDCap will be sent to participants weekly. The MRAES-MBP scale represents the ten most common adverse events and those most highly associated with negative impacts on functioning, in the context of a mindfulness-based program, specifically: signs of hyperarousal (anxiety, perceptual hypersensitivity, traumatic re-experiencing, emotional lability, insomnia), hypo-arousal/blunting (anhedonia, depersonalisation), executive dysfunction and social disengagement [48]. If a participant reports experiencing distress that is impairing their ability to function, they will receive a phone call from our MBSR program teacher, who is an experienced Medical Doctor with extended skills in psychological medicine and a special interest in mindfulness-based interventions, complex trauma and the psychological aspects of chronic pain. Depending on the severity of the distress and the level of impact on functioning, the meditation practices may need to be adjusted or discontinued, or the participant may be advised to discontinue the intervention altogether. Where appropriate, the participant will be referred to the psychologist at the RPAH or CRGH pain management centres.

### Qualitative study

#### Acceptability, and barriers and facilitators of engagement

Qualitative data, collected through in-depth interviews, will explore participants’ experiences with the MBSR program, focusing on barriers and facilitators to engagement and the acceptability of the intervention. Qualitative research is essential for capturing the lived experiences of participants, offering insights that quantitative measures alone cannot provide. By examining participants’ perspectives, we can identify key factors influencing participation, refine the intervention to enhance accessibility and adherence, and inform future implementation strategies [34]. The investigators conducting these in-depth interviews will be independent from the MBSR program. Additionally, the interviewees will not know the investigators conducting these in-depth interviews. All interviews will be audio-recorded and transcribed verbatim.

### Sample size

#### Pilot RCT

Decisions about sample size in pilot studies can be challenging as a variety of approaches exist [49–52]. Some researchers suggest that pilot studies should include at least 30 participants to provide a reliable estimate of the standard deviation, which is essential for calculating the sample size of a future full-scale trial [51]. Based on these guidelines, we plan to recruit 32 participants (approximately 16 per group) for this pilot study. This represents 9% of the estimated sample size for the full trial, which is the minimum threshold recommended by Cocks and Torgerson (2013) [50]. This sample size provides >80% confidence in determining whether to proceed with a full trial, aligning with feasibility objectives. The calculation is based on an estimated effect size difference for the full trial, using a confidence interval approach [50]. A key feasibility outcome is the retention rate, which we anticipate to be 80%. Given our sample size (*N*= 32), this can be estimated to be within approximately ±15% with 95% confidence, meaning that around 26 participants are expected to complete the study [39]. However, we acknowledge that this estimate is provisional and will be refined based on feasibility data collected from the pilot study, including actual retention rates, standard deviation estimates, and adherence metrics.

#### Qualitative study

In qualitative research, the sample size needs to enable rich and diverse data to be collected to provide valuable insights about the topic of interest and to be sufficient to generate an in-depth understanding of it [53]. Based on this information, we have estimated that around 12 to 15 participants may be necessary to reach the qualitative purposes of this study.

### Data analysis

#### Pilot RCT

Baseline demographics will be described using means and percentages and their standard deviations. Primary feasibility outcomes will be analysed using percentages (proportions) and 95% confidence intervals (CIs). Patient-reported outcome measures—including physical function, anxiety, depression, fatigue, sleep disturbance, ability to participate in social roles and activities, pain interference, pain intensity, chronic pain acceptance and mindfulness adherence—will be analysed using between-group comparisons based on intention-to-treat principles Given the exploratory nature of this pilot study, we will report mean differences with 95% confidence intervals to describe potential trends and variability in the data. Quantitative analysis will focus on estimating effect sizes and variability, which will help guide the design and sample size calculation of a future full-scale trial. This approach aligns with best practices for pilot studies, ensuring that the findings are used primarily to inform the feasibility and refinement of the intervention [54].

#### Qualitative study

Qualitative interview data will be analysed using reflexive thematic analysis [55], with the assistance of NVivo software. The Coordinating Principal Investigator and the interviewer will jointly conduct the thematic analysis of the transcripts supervised by an experienced qualitative researcher. Contextual themes will be identified in the data and supported by quotes. All members of the research team will provide input into the results and will be encouraged to engage in reflexivity as part of the analytical process (i.e. reflect on their reactions to the data, how their positionality may influence their interpretation, their motivations to engage in such research, and more). Rigour will be guided by Braun and Clarke’s recommendations for reflexive thematic analysis [55]. The qualitative findings will be integrated with the quantitative results to provide a richer understanding of feasibility outcomes, such as adherence rates and participant-reported experiences. Thematic analysis of the qualitative data will be conducted after quantitative analysis, allowing for triangulation of findings and identification of factors that may influence intervention engagement and effectiveness [55].

### Ethics

This pilot RCT includes key methodological features to minimise bias in clinical trials, such as randomisation, concealed allocation, specification of eligibility criteria, blinded outcome assessment, blinded analysis, and intention-to-treat analysis. Data will be stored in spreadsheets and transferred to appropriate statistical software for analysis by an investigator blinded to group allocation. Spreadsheets will be regularly scrutinised for omissions and errors. Data will be stored and accessed as per the ethics requirements.

We will adhere to the Australian Code for the Responsible Conduct of Research for the storage and archiving of data. Data will be retained for 5 years with restricted access (the research data and/or metadata cannot be shared). All data files (e.g. audiotapes, transcripts) will be stored on a secure server at The University of Sydney, with access restricted to the study investigators. Data files will be coded. After 5 years of storage, the data will be securely destroyed. Electronic data will be deleted, and any paper copies will be shredded.

A Data Safety Monitoring Board (DSMB) will be convened to oversee this study. The board will be comprised of three members: an Associate Professor who is a psychologist, an Associate Professor who is a physician, and a senior physiotherapist who is the Director of Allied Health Services at one of the Sydney Local Health Districts and Co-Chair of the Musculoskeletal Network at the Agency of Clinical Innovation. The DSMB will have an initial meeting before the study commences. In this initial meeting, the DSMB will review the role and functioning of the DSMB, discuss the format and content of the DSMB reports and review scientific and ethical issues relating to the design and conduct of the trial. The DSMB will meet again at 4 weeks after the MBSR program commences and at the end of the 8-week program.

## Discussion

Chronic musculoskeletal pain is a global health challenge that causes a significant burden to individuals and society [1]. Despite continuous advancements in treatment approaches, the prevalence and impact of chronic musculoskeletal pain continue to rise, making it a public health priority [1]. This pilot study lays the groundwork for assessing the feasibility, acceptability, and potential efficacy of integrating an online MBSR program into the Australian public healthcare system, thereby offering hope for a more equitable approach to managing this highly prevalent condition. Despite the evidence supporting MBSR’s efficacy for chronic pain [21], MBSR remains largely inaccessible to patients with chronic musculoskeletal pain conditions within the Australian public healthcare system. Current MBSR programs in Australia are predominantly offered as private courses, often associated with high costs, thus excluding a substantial portion of patients.

Online MBSR programs have become a convenient and accessible alternative to traditional in-person training [25]. Our study will explore the feasibility of offering an online MBSR program to individuals on the waitlists of two pain management centres at two major metropolitan hospitals in Sydney, NSW, Australia. Our feasibility outcomes, such as our recruitment and retention rates, will explore participants’ willingness to engage with this mind-body approach. Our findings will also demonstrate which individuals with chronic musculoskeletal pain are receptive to online interventions and are willing to explore new avenues for pain management. While the primary focus of this pilot study is feasibility and acceptability, preliminary efficacy regarding patient-reported outcomes, including pain, disability, mood, self-efficacy, pain catastrophising, and quality of life, will also provide valuable insights. While the results of the patient-reported outcomes should be interpreted with caution due to the small sample size and the preliminary nature of this study, this process will be insightful as it will demonstrate the feasibility of our data collection methods. Our skilled MBSR teacher will be able to guide participants through potential challenges and ensure their safety and well-being during the program. Nevertheless, adverse events will be monitored closely, addressing a gap in the literature regarding the reporting of adverse events in mindfulness trials.

The qualitative component of this study will provide valuable insights into participants’ experiences, identifying both barriers and facilitators to engaging with the MBSR program. Understanding these factors is critical for refining the online intervention and optimising its acceptability and delivery in future trials and, ultimately, within the healthcare system.

This pilot study will serve as a foundation for potential future research that integrates MBSR into health services. Building on the lessons learned and insights gained from this study, a fully powered clinical trial may be designed and implemented, with a more extensive and diverse participant pool, to provide robust evidence regarding the effectiveness of online MBSR for chronic musculoskeletal pain in the Australian healthcare system context [30]. As we move forward, efforts to reduce health disparities and improve the quality of life for individuals with chronic musculoskeletal pain should continue to be a priority.

### Study limitations

While this pilot study aims to assess the feasibility of an online MBSR intervention for individuals with chronic musculoskeletal pain, some limitations should be acknowledged. Selection bias may arise due to the requirement for English proficiency and internet access, potentially excluding individuals from lower socioeconomic backgrounds. This limitation will be explicitly discussed in the study’s findings interpretation, and future research should explore strategies to enhance accessibility, such as translated materials and mobile-based adaptations. The small sample size limits generalisability, as pilot studies are not powered to detect definitive treatment effects. Differences in engagement between online and in-person MBSR programs may also impact adherence, as digital interventions often experience higher attrition rates [56]. To mitigate this, we will implement structured reminders, participant check-ins, and engagement tracking. Additionally, expectation-related bias may influence adherence, as participants’ perceptions of treatment effectiveness can shape engagement levels [57]. To address these challenges, both groups will receive clear information on the exploratory nature of the study and its contribution to chronic pain research. Despite these limitations, this study will generate valuable feasibility data to refine the intervention and inform a larger-scale trial.

## Supplementary Information

Supplementary Material 1. SPIRIT 2013 Checklist: Recommended items to address in a clinical trial protocol and related documents

## Data Availability

Data will be made available on reasonable request.
